# Generation and characterization of monoclonal antibodies against the N-terminus of alpha-2-antiplasmin

**DOI:** 10.1371/journal.pone.0196911

**Published:** 2018-05-03

**Authors:** Shiraazkhan Abdul, Miet Peeters, Els Brouwers, Joyce J. M. C. Malfliet, Frank W. G. Leebeek, Paul J. Declerck, Dingeman C. Rijken, Shirley Uitte de Willige

**Affiliations:** 1 Department of Hematology, Erasmus University Medical Center Rotterdam, Rotterdam, the Netherlands; 2 Laboratory for Therapeutic and Diagnostic Antibodies, Department of Pharmaceutical and Pharmacological Sciences, KU Leuven, Leuven, Belgium; Duke University School of Medicine, UNITED STATES

## Abstract

Around 70% of circulating alpha-2-antiplasmin (α2AP), the main natural plasmin inhibitor, is N-terminally cleaved between residues Pro12 and Asn13 by antiplasmin-cleaving enzyme. This converts native Met-α2AP into the more potent fibrinolysis inhibitor Asn-α2AP. The Arg6Trp (R6W) polymorphism affects the N-terminal cleavage rate of Met-α2AP in a purified system, with ~8-fold faster conversion of Met(R6)-α2AP than Met(W6)-α2AP. To date, assays to determine N-terminally intact Met-α2AP in plasma have been limited to an ELISA that only measures Met(R6)-α2AP. The aim of this study was to generate and characterize monoclonal antibodies (mAbs) against Met(R6)-α2AP, Met(W6)-α2AP and all α2AP forms (total-α2AP) in order to develop specific Met(R6)-α2AP and Met(W6)-α2AP ELISAs. Recombinant Met(R6)-α2AP, Met(W6)-α2AP and Asn-α2AP were expressed in Drosophila S2 cells. Using hybridoma technology, a panel of 25 mAbs was generated against a mixture of recombinant Met(R6)-α2AP and Met(W6)-α2AP. All mAbs were evaluated for their specific reactivity using the three recombinant α2APs in one-site non-competitive ELISAs. Three mAbs were selected to develop sandwich-type ELISAs. MA-AP37E2 and MA-AP34C4 were selected for their specific reactivity against Met(R6)-α2AP and Met(W6)-α2AP, respectively, and used for coating. MA-AP15D7 was selected for its reactivity against total-α2AP and used for detection. With the novel ELISAs we determined Met(R6)-α2AP and Met(W6)-α2AP levels in plasma samples and we showed that Met(R6)-α2AP was converted faster into Asn-α2AP than Met(W6)-α2AP in a plasma milieu. In conclusion, we developed two specific ELISAs for Met(R6)-α2AP and Met(W6)-α2AP, respectively, in plasma. This will enable us to determine N-terminal heterogeneity of α2AP in plasma samples.

## Introduction

Alpha-2-antiplasmin (α2AP), a member of the serine protease inhibitor (SERPIN) superfamily, is the main natural inhibitor of the fibrinolytic enzyme plasmin [1–3]. Native α2AP is secreted as an approximately 67-kDa single-chain glycoprotein of 464 amino acids, containing 11–14% carbohydrate, with a methionine (Met) as its N-terminus (Met-α2AP) [4]. α2AP is a heterogeneous protein possessing unique N- and C-terminal ends of which the N-terminus is involved in α2AP incorporation into a clot and the C-terminus in the initial interaction of α2AP with plasmin(ogen), as we recently extensively reviewed [5].

In the circulation α2AP undergoes both N-and C-terminal modifications, which significantly alter its inhibitory activity. In approximately 65% of circulating α2AP the C-terminus is intact so it can bind to plasmin(ogen) (plasminogen-binding form of α2AP, PB-α2AP), whereas the remainder (~35%) has lost its ability to bind plasminogen (non-plasminogen-binding form of α2AP, NPB-α2AP) [6–9]. Approximately 70% of circulating α2AP is N-terminally cleaved by antiplasmin-cleaving enzyme (APCE) between residues Pro12 and Asn13, converting native Met-α2AP into the more potent fibrinolysis inhibitor Asn-α2AP [10]. APCE is also known as a soluble, circulating derivative of fibroblast activation protein [11]. A glutamine residue in the N-terminus of α2AP (Gln14) serves as a substrate for activated Factor XIII (FXIIIa) leading to crosslinking and incorporation of α2AP into a fibrin clot [12]. This crosslinking reaction is more efficient when the N-terminus is removed, since Asn-α2AP has been shown to crosslink 13 times more quickly into fibrin than Met-α2AP [10]. Crosslinking of α2AP into fibrin clots results in resistance to fibrinolysis, especially when clots retract [13].

A genetic variation in the *SERPINF2* gene, the gene coding for human α2AP, has been described previously [14–16]. Christiansen *et al*. showed that the Arg to Trp polymorphism of codon 6 (R6W) in the N-terminus of α2AP is functionally significant, as it affects the conversion of Met-α2AP to Asn-α2AP by APCE, and thereby the rate of α2AP incorporation into fibrin [17]. They showed in a purified system that Met(R6)-α2AP was cleaved approximately 8 times more quickly by APCE than Met(W6)-α2AP, relating the polymorphism to the percentage of Met-α2AP in plasma, with the highest percentage Met-α2AP in W6 homozygote individuals. Methods for the measurement of N-terminal variation of α2AP will be useful in order to understand the role of α2AP N-terminal variation in crosslinking and thrombotic disease.

To date, assays to specifically determine N-terminally intact Met-α2AP in plasma have been limited to an ELISA that can only measure Met(R6)-α2AP, as we previously described [18]. The polyclonal antibody used in this ELISA lacked reactivity against Met(W6)-α2AP, meaning that only R6 homozygote individuals could be analyzed. With an allele frequency of 81% for the R allele, on average 66% of study samples can be measured with this ELISA, resulting in loss of power. Therefore, in the current study we generated and characterized monoclonal antibodies (mAbs) with selective reactivity against Met(R6)-α2AP and Met(W6)-α2AP, to improve the assessment of N-terminal variation of α2AP.

## Materials and methods

### Plasma samples

α2AP-depleted plasma was obtained from Affinity Biologicals (Ancaster, Ontario, Canada).

Normal pooled plasma was prepared from citrated apheresis plasma (Sanquin blood bank, Rotterdam, The Netherlands) from 5 healthy donors. Pooled plasma from R6 homozygote individuals (R6 pooled plasma) or W6 homozygote individuals (W6 pooled plasma) was prepared by pooling 100 μl plasma samples from 18 and 17 previously genotyped control subjects of the ATTAC study population, respectively [19]. Genotyping of the α2AP polymorphism R6W (rs2070863) was performed by TaqMan assay [18]. Both plasma pools had a similar α2AP activity in a chromogenic substrate assay.

### Production of recombinant α2AP

Generation of recombinant Met(R6)-α2AP and recombinant Asn-α2AP was performed as described previously [20]. The wildtype construct which results in recombinant Met(R6)-α2AP was used as a template and exposed to a mutagenesis procedure for the production of recombinant Met(W6)-α2AP.

### Generation of mAbs and screening of hybridomas

mAbs against α2AP were produced in-house as described by Galfré and Milstein [21]. A mixture of recombinant Met(R6)-α2AP and Met(W6)-α2AP was used as antigen. The immunization, cell fusion and screening procedure have been performed as described previously [22]. Briefly, BALB/c mice were immunized by subcutaneous injection of 10 μg α2AP in complete Freund’s adjuvant, followed two weeks later by intraperitoneal injection of 10 μg α2AP in incomplete Freund’s adjuvant. After an interval of at least six weeks, the mice were boosted intraperitoneally with 10 μg α2AP in saline on days four and two before the cell fusion. Spleen cells were isolated and fused with myeloma cells using polyethylene glycol. After selection, the supernatants were screened for specific antibody production with one-site non-competitive ELISAs using microtiter plates coated with recombinant Met(R6)-α2AP, Met(W6)-α2AP or Asn-α2AP. The bound immunoglobulins were detected with horseradish peroxidase (HRP)-conjugated rabbit antimouse IgG. Positive clones were cultivated and the IgG fraction of the mAbs was purified from the culture supernatants by affinity chromatography using a ProSep-vA Ultra column (Merck Millipore, Darmstadt, Germany). HRP-conjugated mAbs were produced as described by Nakane and Kawaoi [23]. Determination of antibody isotype was carried out using the IsoStrip Mouse Monoclonal Antibody Isotyping kit (Roche Diagnostics, Vilvoorde, Belgium).

All mouse experiments were carried out after prospective approval of the KU Leuven Ethical Committee for Animal Experimentation (ECD) (Approval Number: P/055/2015). Anesthesia of the mice was performed using sevoflurane.

### Characterization of selected mAbs directed against Met-α2AP by one-site non-competitive ELISA

Purified mAbs were tested for their specific reactivities with α2AP by one-site non-competitive ELISA. 96-well microtiter plates (Nunc A/S, Roskilde, Denmark) were coated with 2 μg/ml (200 μl per well in PBS containing 137 mM NaCl, 2.7 mM KCL, 6.5 mM Na_2_HPO_4_ and 1 mM KH_2_PO_4_ (pH 7.4)) of recombinant Met(R6)-α2AP, Met(W6)-α2AP or Asn-α2AP for 24 hours at 4°C. Non-specific sites were blocked with 200 μl 1% bovine serum albumin (Sigma, Steinheim, Germany) in PBS and incubated for two hours at RT. The plates were washed four times with 200 μl 0.002% Tween-80 in PBS (PBST). Subsequently, 180 μl of serial dilutions (100–0.0001 μg/ml) of purified mAbs in PBST containing 0.1% BSA were added to the wells and incubated for two hours at RT. After four rounds of washing with 200 μl PBST, HRP-conjugated polyclonal rabbit anti-mouse IgG (2 mg/ml) (Sigma) was added at an optimized dilution of 1:10000 and incubated for two hours at RT. Following four washes with 200 μl PBST, the enzymatic activity was determined using o-phenylenediamine (Acros Organics, Geel, Belgium) in a solution containing 0.1 M sodium citrate and 0.2 M di-sodium phosphate and H_2_O_2_ (Merck, Darmstadt, Germany) and the reaction was stopped after 45 minutes by adding 50 μl of 4 M H_2_SO_4_. The absorbance was read at 492 nm on a ELx808 Absorbance Microplate Reader (BioTek Instruments, Winooski,VT).

Relative cross-reactivities of the selected mAbs with Met(W6)-α2AP or Met(R6)-α2AP were calculated by dividing the mAb concentrations at which half-maximal mAb binding occurred and by multiplying this ratio by 100.

### Reactivity of monoclonal antibodies on Western blot

To investigate the reactivities of the mAbs by Western blot, recombinant Met(R6)-α2AP, Met(W6)-α2AP or Asn-α2AP in TBS were boiled for five to ten minutes at 95°C in the presence of XT Sample Buffer and XT reducing agent (BioRad, Richmond, CA) and resolved on a 10% SDS-polyacrylamide gel (300 ng/lane). After electrophoresis the proteins were transferred to a nitrocellulose membrane using a PowerPac™ HC power supply (BioRad) with transfer buffer (25 mM Tris, 192 mM glycine (pH 8.3) and 20% methanol) at 100V constant voltage for one hour. After protein transfer, non-specific sites on the nitrocellulose membrane were blocked with 5% milk in PBS, pH 7.4 followed by three washings in PBS with 0.1% Tween-20. Post-blocking, the blots were incubated with selected purified mAbs (800 ng/ml) in PBS with 0.1% Tween-20 for 18 hours under constant motion at 4°C. After three washes with PBS with 0.1% Tween-20, IRDye^®^ 800CW donkey anti-mouse secondary antibodies (1 mg/ml) (Lincoln Nebraska,USA) were added at an optimized dilution of 1:10000 and incubated for one hour at RT. Following three washes with PBS, the blots were scanned in the 800 nm channel of an Odyssey^®^ Imaging System (Lincoln Nebraska,USA).

### Development of Met(R6)-α2AP and Met(W6)-α2AP sandwich-type ELISAs

Three selected mAbs (MA-AP37E2 for Met(R6)-α2AP, MA-AP34C4 for Met(W6)-α2AP and MA-AP15D7 for all α2AP variants (total-α2AP)) were used to develop sandwich-type ELISAs. MA-AP15D7 was HRP-conjugated to function as detection antibody. 96-well microtiter plates (Nunc A/S, Roskilde, Denmark) were coated with 2 μg/ml of MA-AP37E2 or MA-AP34C4 (110 μl per well in 0.05 M carbonate buffer (pH 9.6)) overnight at 4°C. Non-specific sites were blocked with 150 μl 1% BSA in PBST for one hour at RT and plates were washed once with PBST. Subsequently, serial dilutions (1000–15.6 ng/ml) of recombinant Met(R6)-α2AP, Met(W6)-α2AP or Asn-α2AP with 0,1% BSA in PBST were added. Plates were incubated for two hours at RT followed by four rounds of washing with PBST. Next, MA-AP15D7-HRP (2 mg/ml) was added and incubated for one hour at RT. For the Met(R6)-α2AP ELISA MA-AP15D7-HRP was diluted 8000 times and for the Met(W6)-α2AP ELISA MA-AP15D7-HRP was diluted 2000 times. Following four washes with PBST, enzyme activity was determined using 3,3′,5,5′-tetramethylbenzidine (Sigma) as substrate, and the reaction was stopped after ten minutes by adding 100 μl per well of 1 M H_2_SO_4_. The absorbance was read at 450 nm on a Victor^3^ 1420 multilabel plate counter (Perkin Elmer, Waltham, MA, USA).

For the analysis of native Met-α2AP in plasma, serial dilutions of plasma samples (normal pooled plasma, R6 pooled plasma, W6 pooled plasma and α2AP-depleted plasma) were added. The assays were performed using plasma dilutions of 1:40–1:2560 for the Met(R6)-α2AP ELISA and plasma dilutions of 1:5–1:320 for the Met(W6)-α2AP ELISA. The responses of the different plasma samples in the ELISAs are expressed as the mean result of triplicate measurements.

For the calculation of Met(R6)-α2AP and Met(W6)-α2AP concentrations in plasma samples we took the following in account: (1) normal pooled plasma of healthy individuals contains 70 μg/ml α2AP [5], which is an average value (normal range 45–100 μg/ml), and which refers to total α2AP, including both Met-α2AP and Asn-α2AP, (2) our ELISAs only detect Met-α2AP (3), W6 pooled plasma contains 56.4% Met-α2AP and R6 pooled plasma contains 23.6% Met-α2AP [17]. This implied that R6 pooled plasma contains 16.5 μg/ml Met(R6)-α2AP and W6 pooled plasma contains 39.5 μg/ml Met(W6)-α2AP. Standard curves of R6 pooled plasma and W6 pooled plasma were used for calibration in order to determine Met(R6)-α2AP and Met(W6)-α2AP concentrations in plasma samples. Standard curves were fitted by means of a non-linear regression model using Graphpad Prism (version 5.01).

### Variation of Met(R6)-α2AP and Met(W6)-α2AP

Variation of Met(R6)-α2AP and Met(W6)-α2AP in R6 homozygote individuals (n = 18) and W6 homozygote individuals (n = 17) was determined by the Met(R6)-α2AP ELISA and Met(W6)-α2AP ELISA, respectively. The assays were performed using plasma dilutions of 1:600 for the Met(R6)-α2AP ELISA and plasma dilutions of 1:20 for the Met(W6)-α2AP ELISA. Normal pooled plasma was used to determine the inter- and intra-assay coefficients of variation (CV) of the ELISAs.

### Conversion of Met-α2AP into Asn-α2AP during incubation of normal pooled plasma

The conversion of Met-α2AP into Asn-α2AP by APCE was assessed by incubating normal pooled plasma at 29°C for 0, 6, 24, 30, 48, 54 and 72 hours followed by analysis in the Met(R6)-α2AP and Met(W6)-α2AP ELISAs.

## Results

### Characterization of monoclonal antibodies

**Fig 1:**
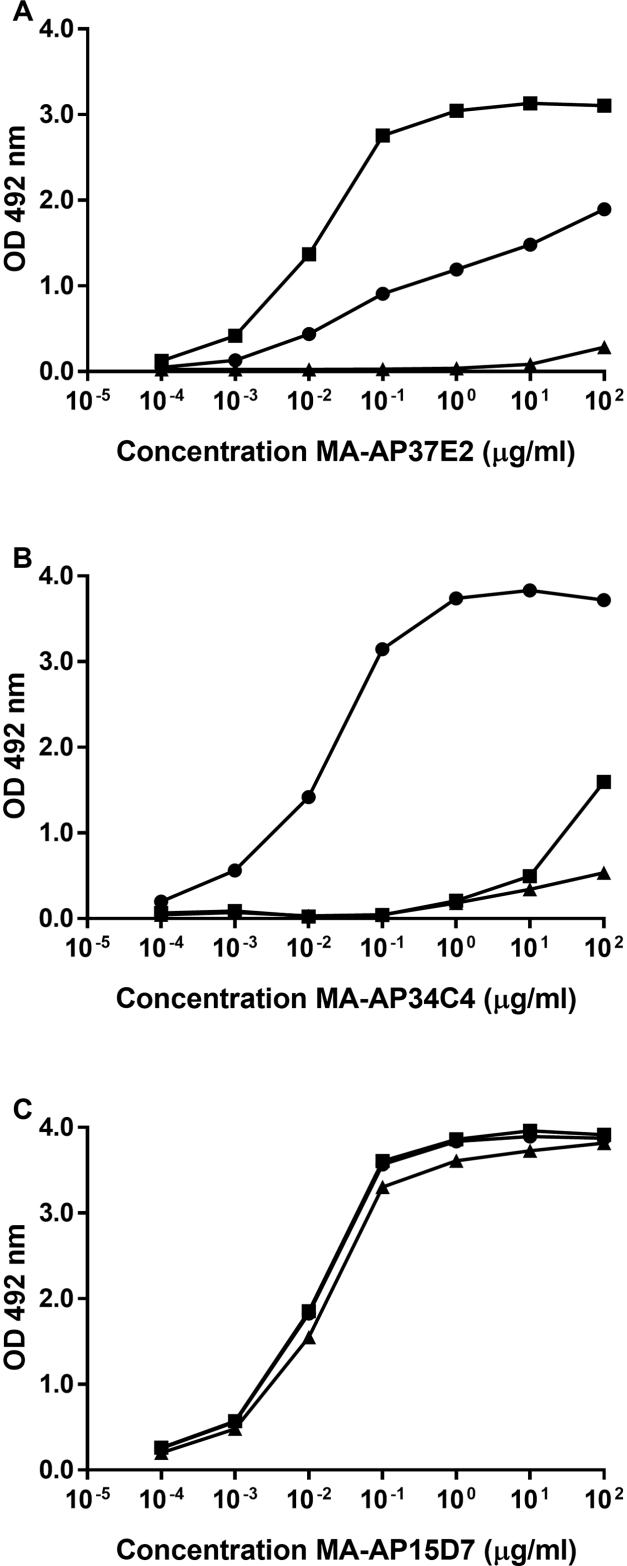
**Reactivity of (A) MA-AP37E2, (B) MA-AP34C4 and (C) MA-AP15D7 with recombinant α2AP by one-site non-competitive ELISA.** 96-well microtiter plates were coated with recombinant Met(R6)-α2AP (■), Met(W6)-α2AP (●) or Asn-α2AP (▲) (2 μg/ml) and serial dilutions (100–0.0001 μg/ml) of MA-AP37E2, MA-AP34C4 or MA-15D7 were added to determine their reactivity. One representative experiment is shown out of three similar experiments. Concentration mAb is shown on a log scale.

In total a panel of 25 mAbs against α2AP was generated of which 8 mAbs were specific for Met-α2AP. For the development of novel Met-α2AP ELISAs we selected three mAbs. MA-AP37E2 and MA-AP34C4 were selected for their relatively specific reactivity against Met(R6)-α2AP and Met(W6)-α2AP respectively (Fig 1A and 1B). MA-AP15D7 was selected for its reactivity against total-α2AP as it reacted equally with Met(R6)-α2AP, Met(W6)-α2AP and Asn-α2AP (Fig 1C). Isotyping of the three selected mAbs revealed that MA-AP37E2 and MA-AP34C4 are IgG1 antibodies with kappa light chains and that MA-AP15D7 is an IgG2b antibody with kappa light chains.

MA-AP37E2 showed 0.1% cross-reactivity with Met(W6)-α2AP and MA-AP34C4 showed 0.01% cross-reactivity with Met(R6)-α2AP (Fig 1A and 1B). MA-AP37E2 showed a small degree of cross-reactivity (estimated to be less than 0.0001%) with Asn-α2AP when MA-AP37E2 was applied at a high concentration (100 μg/ml), while MA-AP34C4 showed some cross-reactivity with Asn-α2AP when it was applied at a concentration from 1 μg/ml to 100 μg/ml. The relative cross-reactivities of both mAbs with Asn-α2AP could not be determined reliably, since half-maximal binding was not reached. Since the mAb concentrations at which cross-reactivity with Asn-α2AP occurred were high, these responses are considered negligible in our assays (see below).

**Fig 2:**
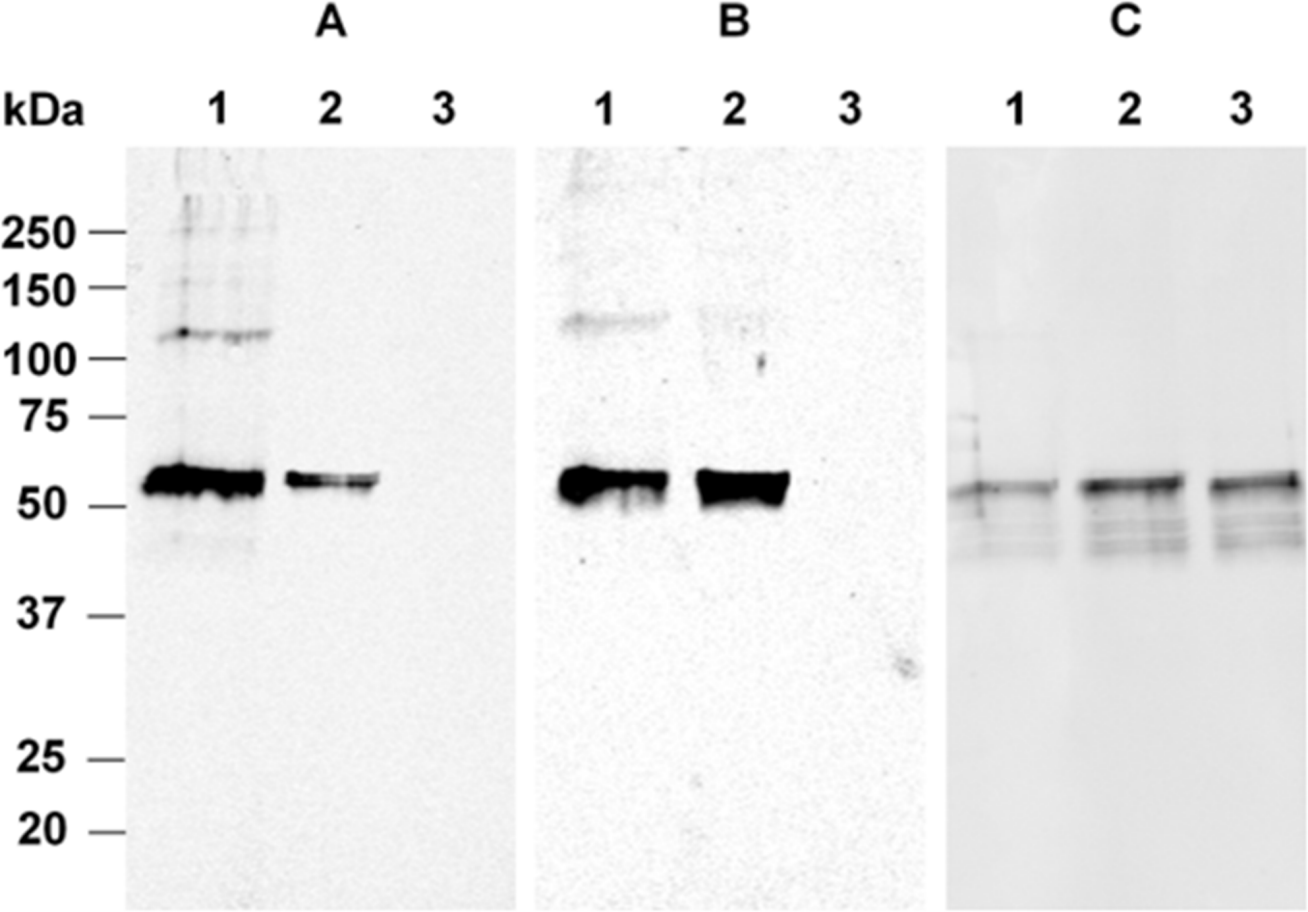
**Reactivity of (A) MA-AP37E2, (B) MA-AP34C4 and (C) MA-AP15D7 with recombinant α2AP on Western blot.** Lane 1: Met(R6)-α2AP (300 ng), lane 2: Met(W6)-α2AP (300 ng), lane 3: Asn-α2AP (300 ng). The migration distances of molecular weight marker proteins are indicated on the left.

In addition to the ELISA analyses, we analyzed the reactivity of the selected mAbs by Western blot. Results showed reactivities of MA-AP37E2, MA-AP34C4 and MA-AP15D7 with protein bands with molecular weights of ~53-kDa, representing recombinant Met(R6)-α2AP, recombinant Met(W6)-α2AP and recombinant Asn-α2AP respectively (Fig 2A, 2B and 2C; S1 Fig). In this approach considerable cross-reactivity was observed. Besides response to Met(R6)-α2AP, MA-AP37E2 showed a weak response to Met(W6)-α2AP (Fig 2A). And MA-AP34C4 showed a similar response to both Met(R6)-α2AP and Met(W6)-α2AP (Fig 2B). Importantly, MA-AP37E2 and MA-AP34C4 did not show any reactivity against recombinant Asn-α2AP (Fig 2A and 2B), indicating their selective reactivity for N-terminally intact Met-α2AP.

### Development of Met(R6)-α2AP and Met(W6)-α2AP sandwich-type ELISAs

**Fig 3:**
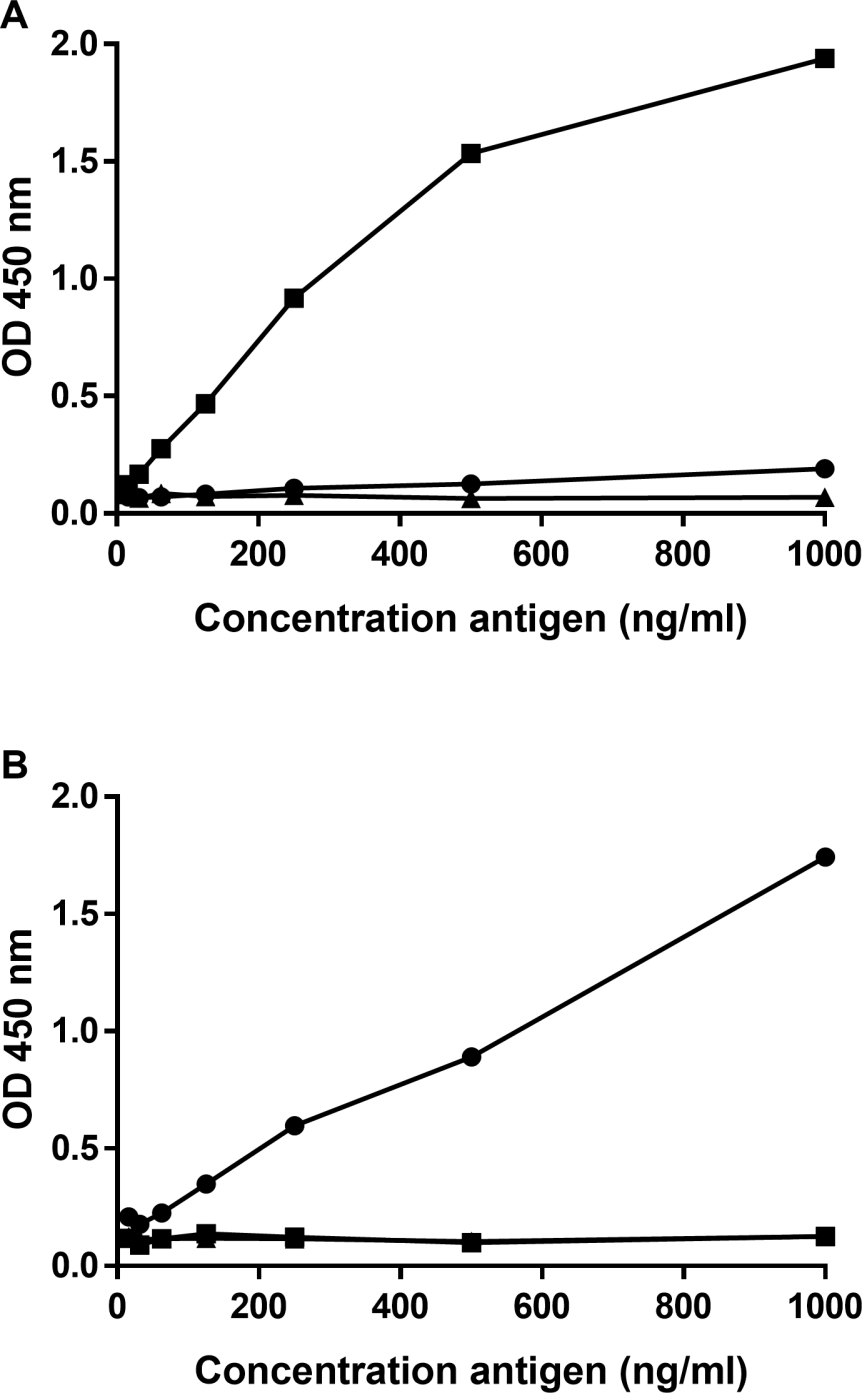
**Reactivity of recombinant α2AP in the (A) Met(R6)-α2AP ELISA and (B) Met(W6)-α2AP ELISA.** 96-well microtiter plates were coated with MA-AP37E2 or MA-AP34C4 (2 μg/ml) and serial dilutions (1000–15.6 ng/ml) of recombinant Met(R6)-α2AP (■), Met(W6)-α2AP (●) or Asn-α2AP (▲) were added. HRP-conjugated MA-AP15D7 was used as a detection antibody. Representative experiments out of three similar experiments are shown.

For the development of sandwich-type ELISAs, MA-AP37E2 (used in the Met(R6)-α2AP ELISA) and MA-AP34C4 (used in the Met(W6)-α2AP ELISA) were applied as coating antibodies and HRP-conjugated MA-AP15D7 as detection antibody. Recombinant Met(R6)-α2AP showed high reactivity in the Met(R6)-α2AP ELISA, whereas virtually no reactivity was observed for recombinant Met(W6)-α2AP and recombinant Asn-α2AP up to a concentration of 1000 ng/ml (Fig 3A). Recombinant Met(W6)-α2AP showed high reactivity in the Met(W6)-α2AP ELISA, whereas virtually no reactivity was displayed for recombinant Met(R6)-α2AP and recombinant Asn-α2AP up to a concentration of 1000 ng/ml (Fig 3B). These results demonstrate the specificities of the Met(R6)-α2AP ELISA and Met(W6)-α2AP ELISA for Met(R6)-α2AP and Met(W6)-α2AP, respectively.

**Fig 4:**
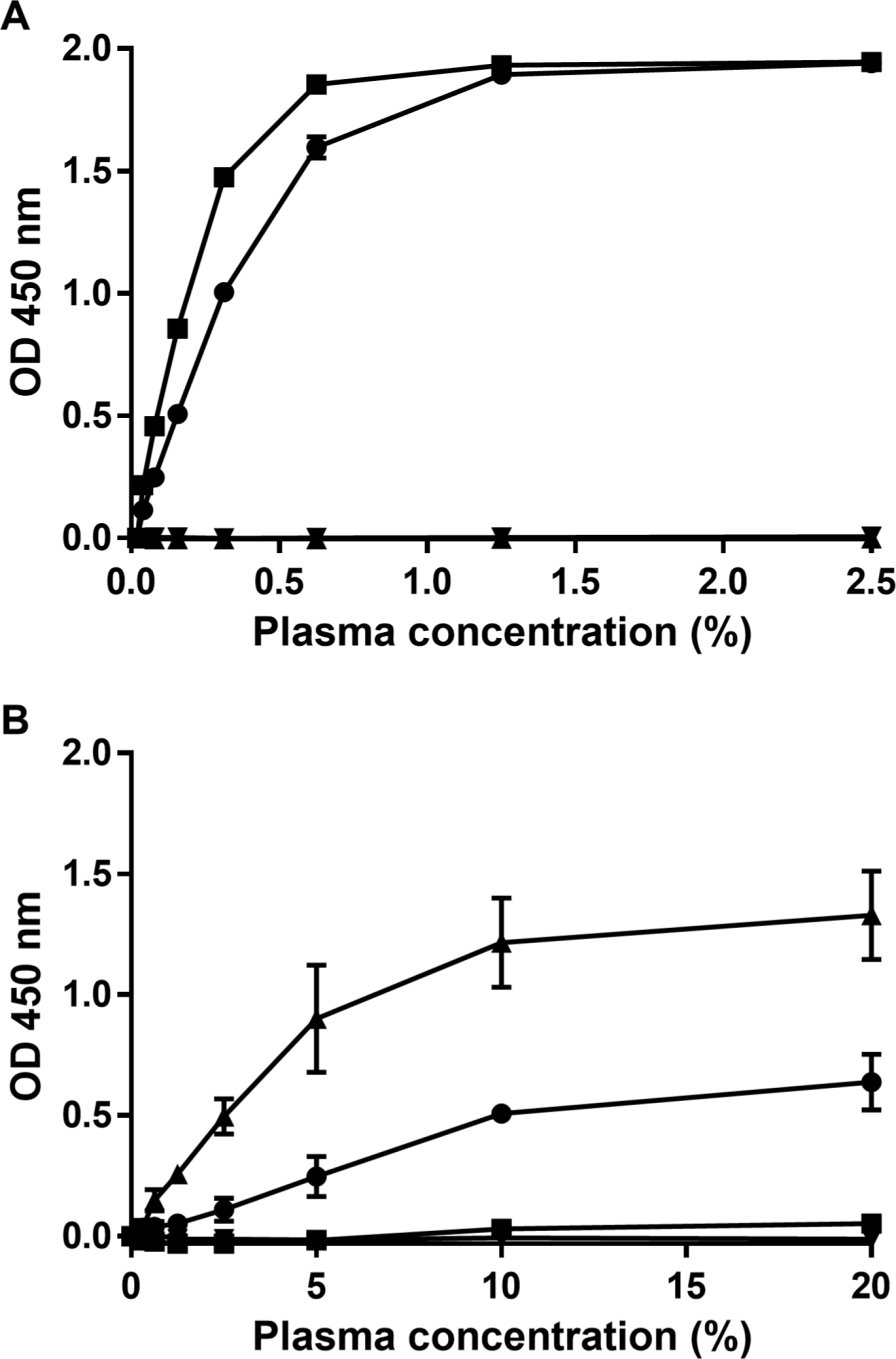
**Reactivity of plasma α2AP in the (A) Met(R6)-α2AP ELISA and (B) Met(W6)-α2AP ELISA.** 96-well microtiter plates were coated with MA-AP37E2 or MA-AP34C4 (2 μg/ml) and varying plasma concentrations (0–2.5% for MA-AP37E2 and 0–20% for MA-AP34C4) of R6 pooled plasma (■), W6 pooled plasma (▲), normal pooled plasma (●) or α2AP-depleted plasma (▼) were tested. Representative experiments out of three similar experiments are shown. Standard deviations of assays performed in triplicate are shown as error bars.

Applying plasma to the novel ELISAs revealed a high reactivity for R6 pooled plasma in the Met(R6)-α2AP ELISA, but no reactivity for W6 pooled plasma. Additionally, W6 pooled plasma showed high reactivity in the Met(W6)-α2AP ELISA, but no significant response was displayed for R6 pooled plasma (Fig 4A and 4B). A matrix effect possibly explains why the responses of the least diluted plasma samples did not further increase in Fig 4B. No reactivity was observed for α2AP-depleted plasma in both ELISAs.

Using the Met(R6)-α2AP and Met(W6)-α2AP ELISAs, we quantified native Met(R6)-α2AP and Met(W6)-α2AP in normal pooled plasma. We found that normal pooled plasma used in the current study contains 6.4 μg/ml Met(R6)-α2AP and 6.9 μg/ml Met(W6)-α2AP.

### Variation of Met(R6)-α2AP and Met(W6)-α2AP

**Fig 5:**
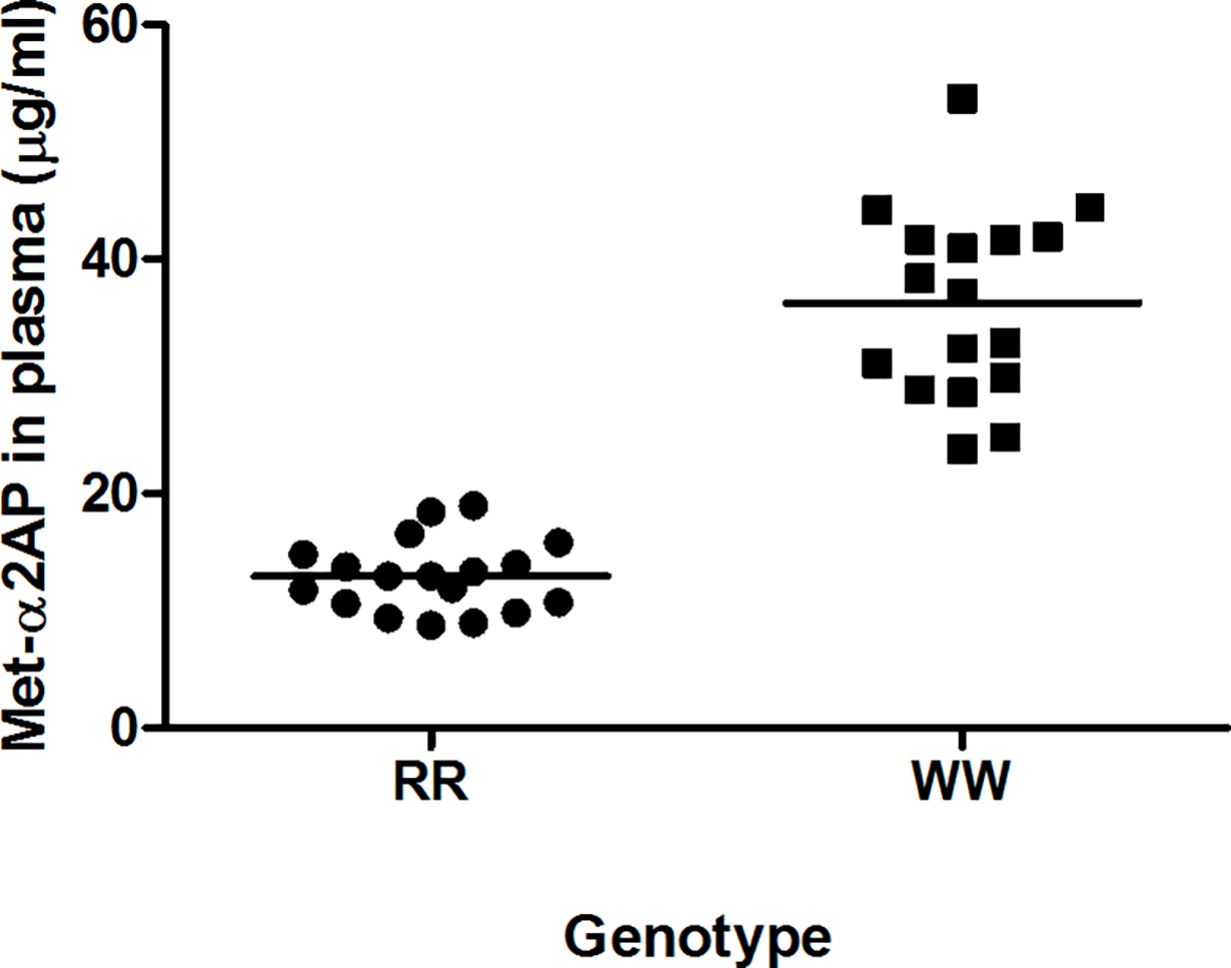
Variation of Met(R6)-α2AP and Met(W6)-α2AP in R6 and W6 homozygote individuals. Concentrations of Met(R6)-α2AP (●*)* as determined by the Met(R6)-α2AP ELISA and concentrations of Met(W6)-α2AP (■) as determined by the Met(W6)-α2AP ELISA. Met(R6)-α2AP levels in 18 R6 homozygote individuals (RR) ranged from 9 to 19 μg/ml with a mean ± sd of 13 ± 3 μg/ml (CV = 24%). Met(W6)-α2AP levels in 17 W6 homozygote individuals (WW) ranged from 24 to 54 μg/ml with a mean ± sd of 36 ± 8 μg/ml (CV = 22%). Bars represent the mean Met-α2AP level.

Plasma samples of R6 and W6 homozygote individuals were analyzed in the Met(R6)-α2AP and Met(W6)-α2AP ELISA, respectively (Fig 5). Met(R6)-α2AP levels in R6 homozygote individuals ranged from 9 to 19 μg/ml with a mean ± sd of 13 ± 3 μg/ml (CV = 24%). Met(W6)-α2AP levels in W6 homozygote individuals ranged from 24 to 54 μg/ml with a mean ± sd of 36 ± 8 μg/ml (CV = 22%). Inter- and intra-assay CV for the Met(R6)-α2AP ELISA were 9.4% and 2.7%, respectively. Inter- and intra-assay CV for the Met(W6)-α2AP ELISA were 16.9% and 4.9%, respectively.

### Conversion of Met-α2AP to Asn-α2AP during incubation of normal pooled plasma

**Fig 6:**
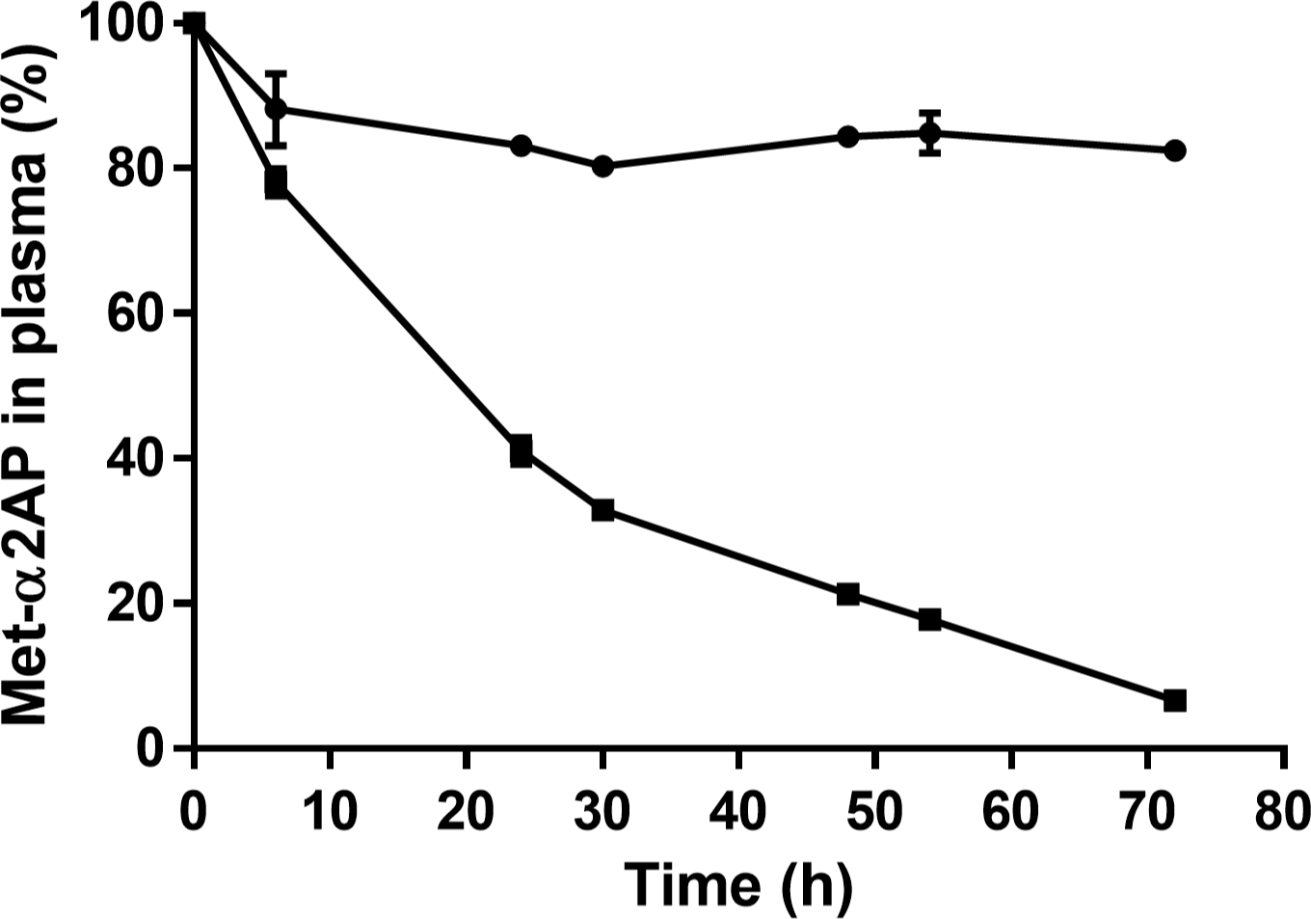
Evaluation of Met-α2AP to Asn-α2AP conversion in normal pooled plasma incubated at 29°C for 72 hours. Percentage of Met(R6)-α2AP (■) and Met(W6)-α2AP (●) as determined by the novel Met(R6)-α2AP and Met(W6)-α2AP sandwich-type ELISAs. The variability of assays performed in duplicate is shown with error bars.

Christiansen et al. showed in a purified system that APCE cleaves Met(R6)-α2AP approximately 8 times more quickly than Met(W6)-α2AP [17]. To study this cleavage in a plasma milieu, we evaluated the conversion of Met-α2AP into Asn-α2AP in normal pooled plasma using the new ELISAs. Over a course of 72 hours, Met(R6)-α2AP was almost completely converted into Asn-α2AP, whereas only approximately 20% of Met(W6)-α2AP was converted (Fig 6). The ELISA results were not sufficiently accurate for a kinetic analysis, however they confirm that Met(R6)-α2AP is converted substantially more quickly into Asn-α2AP by APCE than Met(W6)-α2AP. Furthermore, they show that APCE is active in plasma incubated at 29°C for at least 72 hours.

## Discussion

Current assays to determine N-terminal variation of α2AP in plasma are limited to an ELISA that can only measure Met(R6)-α2AP [18]. Here we describe the development of Met(R6)-α2AP and Met(W6)-α2AP specific ELISAs to overcome this limitation. For this, we generated and characterized novel mAbs against Met(R6)-α2AP (MA-AP37E2) and Met(W6)-α2AP (MA-AP34C4). Additionally, we generated a mAb against total-α2AP (MA-AP15D7) that we used for detection in our ELISAs.

Previously, Christiansen et al. showed that R6 and W6 homozygous individuals have on average 23.6% Met(R6)-α2AP and 56.4% Met(W6)-α2AP, respectively [17]. Based on these percentages, we calculated that R6 homozygous individuals have 16.5 μg/ml Met(R6)-α2AP and that W6 homozygous individuals have 39.5 μg/ml Met(W6)-α2AP, when taking in account that plasma contains 70 μg/ml α2AP. Using our ELISAs we found comparable values, since we determined a mean level of 13 μg/ml Met(R6)-α2AP in R6 homozygote individuals and a mean level of 36 μg/ml Met(W6)-α2AP in W6 homozygote individuals. The Met(R6)-α2AP levels in R6 homozygote individuals were lower compared to Met(W6)-α2AP levels in W6 homozygote individuals. This difference could be explained by 8-fold faster cleavage rate of Met(R6)-α2AP, by APCE, as compared with cleavage of Met(W6)-α2AP [17]. The data of the separate individuals showed that the biological variation in Met(R6)-α2AP (CV = 24%) and Met(W6)-α2AP (CV = 22%) is similar.

Earlier studies have generated and characterized mAbs reacting with distinct parts of the α2AP protein. A mAb against α2AP’s C-terminus has been shown to be useful for the assessment of C-terminal cleavage [24], whereas mAbs against the internal region of α2AP were used for the determination of total-α2AP [25]. Additionally, mAbs that inhibit α2AP activity have been described [26]. A recent study demonstrated that inactivation of α2AP by a mAb results in the dissolution of experimental pulmonary emboli in mice [27]. In this study a similar result was shown after the treatment of pulmonary emboli with recombinant r-tPA. However, in contrast to the r-tPA treatment, the mAb-directed inactivation of α2AP did not show side-effects such as fibrinogen degradation or enhanced experimental bleeding. The authors of the study suggested that modulation of the activity of α2AP by the mAb might have unique therapeutic value in pulmonary embolism. To date, no mAbs have been described as being against the α2AP N-terminus. One of the reasons is that the original preparations of purified α2AP contained only Asn-α2AP [28]. Newer preparations revealed also Met-α2AP [4], while the R6W SNP was only recognized in 2007 [17].

In a previous study on the assessment of α2AP N-terminal variation in plasma, the Met-α2AP ELISA used a polyclonal antibody raised against the first twelve N-terminal amino acids of Met-(R6)-α2AP [18]. This polyclonal antibody only recognized Met(R6)-α2AP and not Met(W6)-α2AP. The current study confirms that the immunological properties of the Met(W6)-α2AP N-terminus differ from those of the Met(R6)-α2AP N-terminus. It is interesting to note that none of the 8 generated Met-α2AP specific antibodies reacted equally well with Met(R6)-α2AP and Met(W6)-α2AP (results not shown). The limitation of specificity of the polyclonal antibody for only Met(R6)-α2AP is solved by our ELISAs as we can now measure both Met(R6)-α2AP and Met(W6)-α2AP.

We used the newly developed ELISAs to show that endogenous Met(R6)-α2AP present in plasma is converted more quickly into Asn-α2AP by APCE compared to Met(W6)-α2AP. This confirms and extends a previous study performed with purified Met(R6)-α2AP and Met(W6)-α2AP by Christiansen et al., who showed that Met(R6)-α2AP is a better substrate for APCE than Met(W6)-α2AP, since Met(R6)-α2AP was cleaved ~8 times more quickly to Asn-α2AP [17]. Possibly, the difference in the reactivity of APCE to either Met-α2AP variant is affected by the same differences in conformational properties between Met(R6)-α2AP and Met(W6)-α2AP N-termini that cause their antigenic properties. Moreover, Met(R6)-α2AP decreased over the course of a 72-hour plasma incubation, indicating that APCE remains active during that time period.

The selective reactivities of MA-AP37E2 and MA-AP34C4 in ELISA were not observed in the Western blot experiments. On the blots we found considerable cross-reactivity from both mAbs. The discrepancy between the ELISA- and Western blot results may partially be explained by our definition of cross-reactivity in ELISA (for instance, the ratio of mAb concentrations at which half-maximal mAb binding occurred in Fig 1) and partially by the conformational status of both Met-α2AP variants, which are different in the ELISA and Western blotting procedures. Incomplete renaturation of Met-α2AP during Western blotting, resulting in a more linear protein, might result in cross-reactivity of MA-AP37E2 with Met(W6)-α2AP and of MA-AP34C4 with Met(R6)-α2AP. This incomplete renaturation should not be present in ELISA, since the native conformation of the proteins is retained. Since Met(R6)-α2AP only differs from Met(W6)-α2AP by one amino acid residue at position 6 (R6W) on a stretch of 12 amino acids, the recognition of a linear amino acid sequence of both proteins by MA-AP37E2 and MA-AP34C4 will be partially similar. However, since a tryptophan is a more complex amino acid than an arginine, this substitution may have consequences for the 3D protein structure of the proteins [17]. To date, there is no 3D protein structure available for the entire N-terminus of α2AP [29].

To summarize, we have described the development of two novel ELISAs for Met(R6)-α2AP and Met(W6)-α2AP in plasma. This will enable us to improve the evaluation of the fibrinolytic potential in plasma samples in various disease states.

## Supporting information

S1 Fig **Original Western blot result showing the reactivity of (A) MA-AP37E2, (B) MA-AP34C4 and (C) MA-AP15D7 with recombinant α2AP.** Lane 1: Met(R6)-α2AP (300 ng), lane 2: Met(W6)-α2AP (300 ng), lane 3: Asn-α2AP (300 ng). α2AP protein bands are visualized in green. The molecular weight marker proteins are visualized in red.
(TIF)
