# Supplementary figures and images for: Generation and characterization of monoclonal antibodies against the N-terminus of alpha-2-antiplasmin
Source: PLoS One. 2018 May 3;13(5):e0196911. doi: 10.1371/journal.pone.0196911 (PMC5933735; doi:10.1371/journal.pone.0196911)

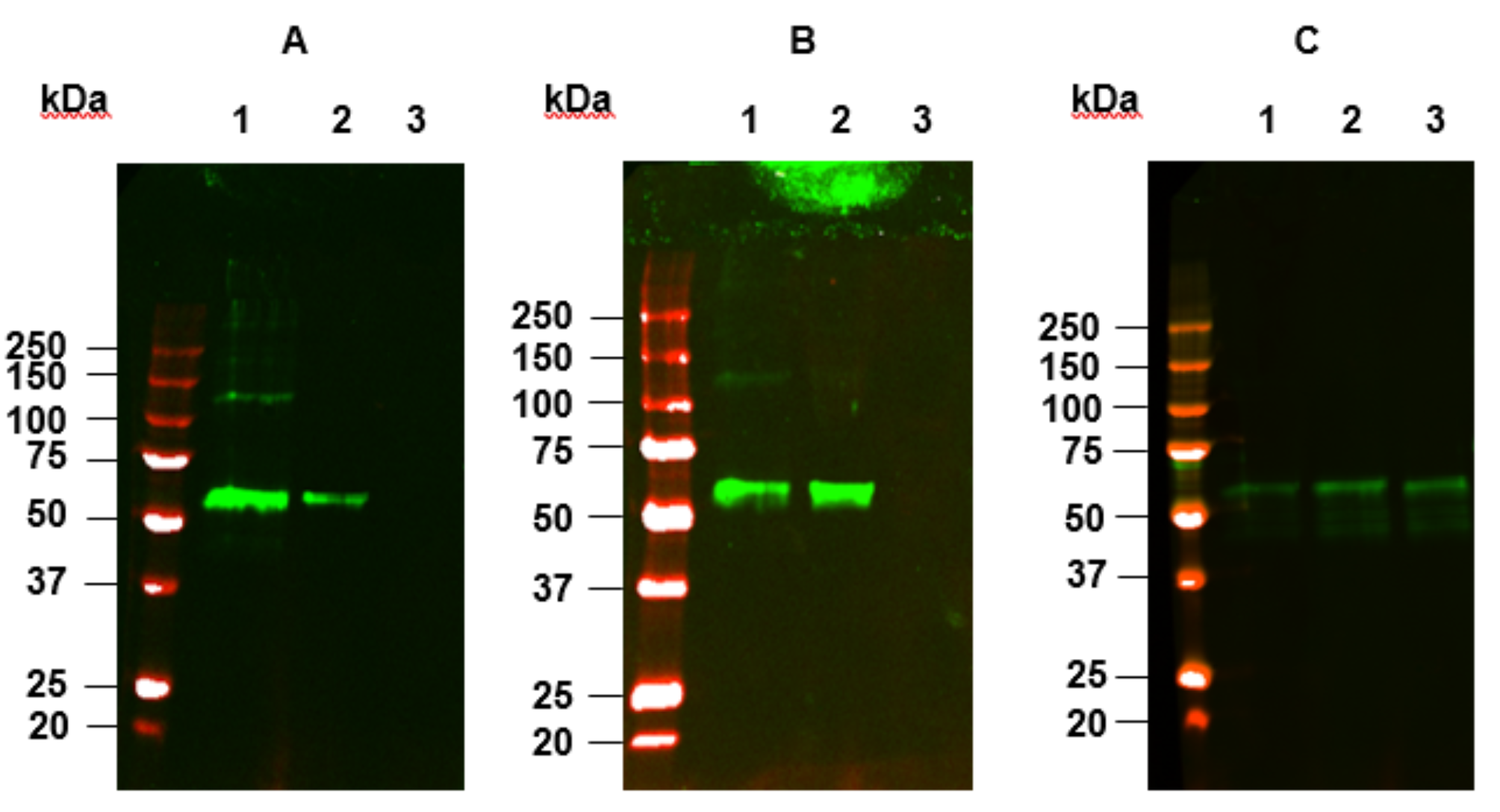

Supplement: S1 Fig — Original Western blot result showing the reactivity of (A) MA-AP37E2, (B) MA-AP34C4 and (C) MA-AP15D7 with recombinant α2AP. Lane 1: Met(R6)-α2AP (300 ng), lane 2: Met(W6)-α2AP (300 ng), lane 3: Asn-α2AP (300 ng). α2AP protein bands are visualized in green. The molecular weight marker proteins are visualized in red. (TIF) [file pone.0196911.s001.tif]
